# Service change and innovation in community end-of-life care during the COVID-19 pandemic: Qualitative analysis of a nationwide primary care survey

**DOI:** 10.1177/02692163211049311

**Published:** 2021-12-17

**Authors:** Sarah Mitchell, Madeleine Harrison, Phillip Oliver, Clare Gardiner, Helen Chapman, Dena Khan, Kirsty Boyd, Jeremy Dale, Stephen Barclay, Catriona R Mayland

**Affiliations:** 1 University of Sheffield, Sheffield, UK; 2 Sheffield Teaching Hospitals NHS Foundation Trust, Sheffield, UK; 3 Patient and Public Involvement Representative, Birmingham, England, UK; 4 University of Edinburgh, Edinburgh, UK; 5 University of Warwick, Coventry, England, UK; 6 University of Cambridge, Cambridge, UK; 7 University of East Anglia Norwich Medical School, UK

**Keywords:** Primary health care, general practice, primary care nursing, palliative care, end-of-life care, COVID-19

## Abstract

**Background:**

Primary healthcare teams (general practice and community nursing services) within the United Kingdom provided the majority of community end-of-life care during COVID-19, alongside specialist palliative care services. As international healthcare systems move to a period of restoration following the first phases of the pandemic, the impact of rapidly-implemented service changes and innovations across primary and specialist palliative care services must be understood.

**Aim:**

To provide detailed insights and understanding into service changes and innovation that occurred in UK primary care to deliver end-of-life care during the first phase of the COVID-19 pandemic.

**Design:**

Cross-sectional online survey. Responses were analysed using descriptive statistics and thematic analysis.

**Setting/participants:**

United Kingdom survey of general practitioners and community nurses, circulated via regional and national professional networks.

**Results:**

A total of 559 valid responses were received from 387 community nurses, 156 general practitioners and 16 ‘other’. Over a third of respondents (*n* = 224; 40.8%) experienced changes in the organisation of their team in order to provide end-of-life care in response to the COVID-19 pandemic. Three qualitative themes were identified: COVID-19 as a catalyst for change in primary palliative care; new opportunities for more responsive and technological ways of working; and pandemic factors that improved and strengthened interprofessional collaboration.

**Conclusion:**

Opportunity has arisen to incorporate cross-boundary service changes and innovations, implemented rapidly at the time of crisis, into future service delivery. Future research should focus on which service changes and innovations provide the most benefits, who for and how, within the context of increased patient need and complexity.

**What is already know about this topic?**

- Primary healthcare teams deliver the majority of end-of-life care in the community, but barriers exist including time pressures, compromised continuity of care and variable access to specialist palliative care services.
- Primary and specialist palliative care services have had to adapt rapidly to meet increased need for end-of-life care in the community during the COVID-19 pandemic.
- There is a stark lack of evidence from previous pandemics to guide service changes and policy for both primary care, specialist palliative care and collaborative working.

**What this paper adds?**

- This paper provides insights into the changes perceived to improve end-of-life care in primary care during the first phase of the COVID-19 pandemic including collaborative working with specialist palliative care colleagues using technology.
- Individual efforts and increased working hours and opportunities for more flexible, responsive working allowed the increased need for end-of-life care in the community to be addressed.
- Shared goals for patient care enabled the relationships between primary and specialist palliative care colleagues.

**Implications for practice, theory, or policy**

- Future models for community end-of-life care should enable the efforts of motivated individuals in primary care, in collaboration with colleagues from specialist palliative care.
- Future research into the relationships between primary and specialist palliative care will enhance future integrated models of palliative and end-of-life care.
- Future research into the use of technology to facilitate collaborative working is an important next step and should inform policy.

## Introduction

Primary healthcare services (general practice, district nursing and community nursing services) play a pivotal role in the delivery of palliative and end-of-life care in the community, in the UK and internationally.^1,2^ Prior to the COVID-19 pandemic, multiple barriers were described to such care, including time and resource pressures, a lack of support for patients and families in the community, inconsistent training and variable access to specialist palliative care.^3^ Tensions have been described in the relationships between primary healthcare teams and specialist palliative care teams, with a lack of clarity around roles and responsibilities.^4–6^

The COVID-19 pandemic has been associated with a significant and sustained increase in the need for community end-of-life care. Primary healthcare services have adapted quickly to new challenges including the use of virtual consultations and the management of new symptom profiles associated with COVID-19.^7–9^ The evidence base for end-of-life care in primary care from previous pandemics is severely lacking, and policy documents for primary care make almost no reference to this area of practice.^10,11^ Consequently there has been little to guide the necessary primary care service changes through the COVID-19 pandemic.

The response of specialist palliative care services to the COVID-19 pandemic has been documented in a multi-national survey that identified a number of service changes including: streamlining, extending and increasing outreach of services, implementing staff wellbeing innovations and using technology to facilitate communication.^12^ Factors enabling change included: collaborative teamwork, staff flexibility, a pre-existing IT infrastructure, pooling of staffing resources and strong leadership. To our knowledge, the only exploration of palliative and end-of-life care delivered by primary care providers has been produced by this team.^11^ From September to October 2020, we conducted a survey of primary health care professionals to understand their experiences of providing end-of-life care during the pandemic. Initial findings of the survey detailed conflicting roles between general practitioners and community nurses. General practitioners reported increased use of remote consultations, whilst community nurses took greater responsibility for the delivery of face-to-face end-of-life care with limited support, resulting in emotional distress.^13^

This paper reports the findings of a further analysis of the survey data, specifically focussed on service changes perceived by primary healthcare professionals to have worked well and the opportunities they afford moving forward. The aims of the analysis were (1) to provide detailed insights into the primary care service and organisational changes that enabled the delivery of end-of-life care in the community during the first phase of the COVID-19 pandemic and (2) to identify opportunities for future service delivery and development.

## Methods

### Study design

A web-based, UK-wide questionnaire survey was considered the most feasible method at the time of the study, during the first phase of the COVID-19 pandemic, to reach a large number of potential participants as quickly as possible. Responses were analysed using descriptive statistics and an inductive thematic analysis. The study design was informed by national^14^ and local patient and public consultation work, including the recruitment of a patient and public involvement member to the study steering group. Ethical approval was granted by the University of Sheffield Research Ethics Committee on 28 July 2020 (reference number: 035508). Reporting was informed by the STROBE checklist.^15^

### Survey instrument

The survey instrument was informed by a rapid review of the existing evidence of the role and response of primary care in end-of-life care during pandemics (conducted by members of this team^11^), the CovPall study of palliative care services^16^ and feedback from the study advisory group. A total of 17 community nurses and general practitioners pre-tested the survey, with feedback leading to minor edits of the questions for clarity and changes to the order of the questions. Closed questions collected demographic details and quantitative data about service changes. Open-ended questions allowed for the collection of in-depth qualitative data about the perceptions and experiences of participants. The survey instrument is provided in full in Supplemental Appendix 1.

### Setting

A link to the survey (a GoogleForm) was circulated via email bulletins, newsletters and social media posts via UK professional networks locally and nationally, including the Royal College of General Practitioners, the Society for Academic Primary Care, the Royal College of Nursing, The Queen’s Nursing Institute and the National District Nursing Network. Data were collected as soon as possible after ethics approval was granted, between 01 September and 16 October 2020. Responses from GPs or community nurses were included, responses from other healthcare professionals were excluded. The aim was to achieve a diverse sample of at least 500 participants (which was considered realistic having previously attained such sample sizes in similar populations^3^).

### Data analysis

Quantitative data were analysed using descriptive statistics using SPSS (version 26). Free-text responses were anonymised and uploaded into NVivo software (QSR international, release 1.4). This analysis was of responses to questions from the survey as follows: (1) whether service changes have occurred, (2) which services were developed, (3) what changes worked well, (4) any innovations respondents would like to see continue beyond COVID-19 and (5) the opportunities respondents perceived to have arisen from these changes. Inductive thematic analysis was undertaken on the qualitative data following an iterative approach. Given the scale of service and behaviour change that occurred over a short timeframe in community end-of-life care, Kurt Lewin’s behaviour change model ‘unfreeze-change-refreeze’ model informed the analysis. This provided a framework to describe the rapid ‘unfreezing’ of the status quo, followed by ‘change’, and consideration of the changes that should be preserved (the ‘refreeze’) into the future.^17,18^ Each data item was coded, and the codes collated into a thematic framework that was grouped into overarching themes. Data analysis was led by MH with 20% of qualitative responses analysed independently by SM and regular discussion of the emerging findings with the wider study team in order to reduce lone researcher bias.^19^

## Results

### Demographics

In total, 563 respondents completed the survey; responses from healthcare professionals other than general practitioners and community nurses were excluded, resulting in 559 valid responses. The sample included: 387 community nurses, 156 general practitioners and 16 unspecified responses (see Table 1 for demographic information). Responses were received from all countries within the United Kingdom; 77.1% were from England. Urban, rural and innercity areas were represented with the most common response being a ‘mixed urban and rural’ area (39.9%):

**Table 1.** Demographic information (*n* = 559).

	*N*	%
What is your role? (*n* = 543)		
Doctor	156	28.7
General practice partner	104	19.1
Sessional general practitioner	45	8.3
Other, for example General practitioner in training	7	1.3
Community nurse	387	71.3
Community staff nurse (registered nurse with degree level training, working in the community)	150	27.6
District nurse (including team leaders) (registered nurse with special training in community care)	159	29.3
Advanced nurse practitioner (registered nurse with Masters level qualification)	32	5.9
Community matron (Senior nurse working with patients with serious long term conditions or complex healthcare needs in the community)	24	4.4
Community healthcare assistant (care professional working under the guidance or supervision of registered nurses)	15	2.8
Nurse consultant (a nurse who has specialised in a specific area of practice, with further academic study, research and extensive clinical experience)	7	1.3
Missing	16	
Which country do you work in? (*n* = 559)		
England	431	77.1
Scotland	65	11.6
Wales	47	8.4
Ireland	16	2.9
Missing	*–*	
What type of area do you work in mainly? (*n* = 556)		
Mixed urban and rural	222	39.9
Urban	179	32.3
Rural	106	19.0
Innercity	49	8.8
Missing	3	

### Quantitative findings

Almost a third of general practitioner respondents (27.9%) reported a change in their working hours. Of these, all reported that this was informal change, including working later into the evenings to provide home visits and the provision of bespoke out-of-hours ‘on-call’ services to care homes at weekends. Of nurse respondents, 36.5% reported changes in working hours including extra hours worked informally, starting shifts early and finishing late to ensure that patients received the care they needed. Formal changes, including planned overtime and changes in shift patterns, were reported by 46.2% of community nurses whose hours of work changed.

Overall, 40.8% of respondents experienced changes in the organisation of the services they worked for orientated to increased need for community end-of-life care, including extension of the hours of community nursing services beyond a 9 am–5 pm service (10.6%). A small percentage of community nurses reported an increase in their level of responsibility for prescribing in end-of-life care (6.4%) and involvement in new services to facilitate carer administration of medications to patients at the end-of-life (6.3%). New provision of an out-of-hours specialist palliative care team on-call service alongside primary care services was reported by only 7.3% of participants. Table 2 provides further details of these changes, including a comparison of doctors and community nurses. Nurses were more likely than doctors to report having cared for patients who had died from confirmed or suspected COVID-19 and reported providing more end-of-life care at home during the pandemic.

**Table 2.** Provision of care for dying patients and details of how services have changed in response during the pandemic (*n* = 559).

	All*		Role**				
	*N*	%	Doctor (*n* = 156)		Community nurse (*n* = 387)		*p*-Value^†^
			*N*	%	*N*	%	
Have you cared for any patients in the community who have died with confirmed (by test) COVID-19? (*n* = 559)							
Yes	296	53.1	68	43.9	221	57.3	0.006
No	261	46.9	87	56.1	165	42.7	
Missing	2		1		1		
Have you cared for any patients in the community who have died with suspected COVID-19 (untested but with clinical symptoms)? (*n* = 554)							
Yes	371	67.0	87	56.1	275	71.6	0.001
No	183	33.0	68	43.9	109	28.4	
Missing	5		1		3		
Have you been involved in providing end-of-life care at home for patients who do not have COVID-19 or suspected COVID-19 through the pandemic? (*n* = 554)							
A lot more than usual	172	31.1	5	3.2	160	41.6	<0.001
A little bit more than usual	150	27.1	35	22.6	112	29.1	
About the same as usual	211	38.1	103	66.5	104	27.0	
A little bit less than usual	13	2.3	9	5.8	4	1.0	
A lot less than usual	8	1.4	3	1.9	5	1.3	
Missing	5		1		2		
Have your working hours changed in order to deliver end of life care during COVID-19? (*n* = 555)							
Yes	189	34.1	43	27.9	141	36.5	0.070
No	366	65.9	111	72.1	245	63.5	
Missing	4		2		1		
Have there been any changes in the organisation of your team in order to provide end of life care during the COVID-19 pandemic? (*n* = 549)							
Yes	224	40.8	50	32.7	167	43.8	0.019
No	325	59.2	103	67.3	214	56.2	
Missing	10		3		6		

* Missing data reported but not included in percentages.

** Sixteen respondents didn’t state their role.

† Fisher’s exact test of the association between survey response and role.

## Qualitative findings

Three interconnected themes related to service changes that were considered beneficial were identified. These are described below:

### Theme 1: COVID-19 as a catalyst for change in primary palliative care

Respondents described the implementation of new policies, protocols and guidelines around a variety of aspects of palliative care, most of which were implemented temporarily in the first instance. Increased flexibility in systems and processes was perceived to have resulted from the ‘*loosening of governance rules*’ and ‘*breaking down local boundaries*’. Changes in the law and national guidance for verification of death processes and completion of cremation forms in England were widely praised and many respondents perceived these temporary changes should not be reversed following the COVID-19 pandemic.

‘Verification of death (VoD) - with regards this process, it has been a big help for both community nurses and also families to be able to verify a patient’s death as this has meant less delays in the process to get undertakers and also more support and assistance to the families as the nurses who were verifying were normally looking after the patient, so the nurses were well known by the families which meant more support was given to them.’ (Respondent 174, District Nurse, England)

Changes in medicines management were described at both an organisational and service level, including upskilling of nursing staff in prescribing and transcribing of medications, the availability of ‘just in case’ medications for all patients in care homes and ‘grab bags’ for symptom control medication by community nursing teams. Changes to national guidance around medicine re-use in care homes and hospices were received positively. A small number of community nurses reported a movement towards family carers being trained to deliver end-of-life medications, which was felt to be particularly useful in rural areas.

‘The NHS pharmacy involvement was a game changer. One local challenge we have is access to end-of-life drugs out of hours in situations where a patient deteriorates before anticipatory prescribing has been possible. Also with the nursing care home there were too many patients with covid-19 and a shortage of some palliative drugs. We could not prescribe “just in case” meds individually and had a limited supply in the practice for in hours use. The pharmacy team designed a ‘grab bag’ for OOH [out of hours] practitioners to take into the home. But then we realised patients sometimes deteriorated so quickly that these drugs were best kept securely in the home so that the nursing home nurses (who had a lot of palliative care experience) could administer if needed.’ (Respondent 48, General Practitioner Partner, Scotland)

The need for increased palliative and end-of-life care in the community was highlighted throughout the responses, with community nurses providing the majority of face-to-face care. In some areas, community nursing teams described extending their hours, as well as setting-up ‘hospital at home’ teams and facilitating newly established urgent hospital discharge processes. Some respondents reported that the visibility of community nursing, and their contribution to end-of-life care, increased amongst other healthcare professionals:

‘Evidencing the value of DN [District Nursing] services in providing generalist palliative and end-of-life care, and growing the reputation and resources of these services as a result.’ (Respondent 404, District Nurse, England)

Many respondents described the opportunities afforded by the changes to primary palliative care services as a consequence of the COVID-19 pandemic. Respondents welcomed the speed and openness to change experienced during the pandemic:

‘Digital technology and remote prescribing was available after a number of two year projects all of a sudden came to fruition’ (Respondent 491, Nurse Consultant, England)

However, there was recognition of the need to reflect on the changes made and best practice for future waves of COVID-19 or a future pandemic.

‘We have been able to review what has occurred with processes over past 6 months and agree what to keep or adjust.’ (Respondent 442, Community Staff Nurse, England)

### Theme 2: Opportunities for more responsive and technological ways of working

More responsive ways of working were facilitated by the increased use of technology during the first phase of the COVID-19 pandemic. These were generally perceived to have worked well. The speed of adoption and willingness to engage with new technologies and other innovations were directly attributed to the pandemic. However, there was variation in respondents’ desire for different changes to be maintained. The widespread reliance on technology caused by the pandemic was perceived to have normalised the use of virtual communication.

‘[the use of technology is] becoming the norm and more accessible and acceptable as a valid form of communication than prior to Covid.’ (Respondent 169, General Practice Partner, England)

The benefits of increased technology use for virtual meetings included increased ability to hold and attend regular interprofessional meetings and fewer follow-up actions after meetings as all relevant parties could attend.

‘Increased MDT [multi-disciplinary team] meetings in my team meaning more chance to discuss patients with medical staff.’ (Respondent 223, Community Staff Nurse, Scotland)

Reduced travel time and being able to start and finish the day from home were also described as benefits.

‘Virtual working has innovated practice, has removed some ties that keep us working from offices and made our care more flexible.’ (Respondent 318, Community Matron, England)

Specific aspects of community end-of-life care that were recognised as having improved due to increased technology use included more regular contact with care home staff, referral processes when electronic systems were adopted across organisations including hospices, and secure email services for sharing patient information. Respondents perceived that there was an opportunity to maintain and build upon the changes that had occurred, particularly in relation to virtual meeting attendance and having mobile devices with access to electronic systems. Other key developments included shared electronic patient records and systems across disciplines and organisations. Further investment for technology infrastructure and mobile devices were identified as vital to enable the changes to be maintained:

‘Time to get adequate investment into digital infrastructure in the community.’ (Respondent 46, General Practice Partner, Scotland)

There were mixed views about the use of technology for patient consultations. Some respondents reported video consultations working well, while others expressed caution about their use in palliative and end-of-life care, particularly advance care planning:

‘I think as a GP [General Practitioner] there are lots of things in general I’d like to continue but when it comes to palliative care a lot of it is very difficult and not optimal if it can’t be done face-to-face.’ (Respondent 74, Sessional General Practitioner, England)

There were positive experiences including the ability to involve carers who did not live close to the patient, joint virtual consultations with colleagues from other teams and virtual ward rounds in care homes.

‘We managed to call lots of patients and get pro-active care plans . . . done - these were not easy conversations over the phone - but almost always patients were happy to describe what they wanted to happen if they became unwell.’ (Respondent 143, General Practice Partner, England)

### Theme 3: Pandemic factors that improved and strengthened interprofessional collaboration

The COVID-19 pandemic drew attention to existing gaps in effective interprofessional communication in end-of-life care, and a willingness to improve collaborative relationships. This was attributed to all healthcare professionals working towards a common goal in response to a crisis situation.

‘Better communication between services. I think this has been highlighted as an issue providing an opportunity to improve it.’ (Respondent 192, Deputy District Nurse Team Leader, England)

New opportunities to ‘open a dialogue’ between professionals from different specialities and break down professional boundaries were described:

‘Opened a dialogue between primary care and SPC [Specialist Palliative Care] about challenges and potential solutions.’ (Respondent 149, Sessional General Practitioner, Scotland)

Most respondents described an opportunity to build and/or strengthen collaborative relationships. This required dedicated healthcare professionals with a ‘can do’ attitude who were willing to pull together, placing confidence and trust in the abilities of other healthcare professionals.

‘Improved professional trust and understanding of specialist palliative care and what this adds to generalist skills and knowledge.’ (Respondent 350, Nurse Consultant, England)

The benefits of improved communication and collaboration identified by respondents included more efficient working and reduced duplication of effort, improved information sharing about patients, speedier discharge from hospital and fewer delays in prescribing. There was a perception that this provided patients with more opportunity to die in the place of their choice. Many respondents hoped these improvements would be maintained:

‘There has been a lot more collaboration between the specialist palliative care nurses and the community nursing team. There needs to be more communication not just during the pandemic, but looking forward into a post-covid world.’ (Respondent 218, Community Staff Nurse, England)

Both general practitioner and community nurse respondents described opportunities for education and training about palliative care being made available during the COVID-19 pandemic. Formal training opportunities were typically provided to healthcare professionals working in the community by hospices or specialist palliative care consultants, accessed virtually via video conferencing, webinars and Project ECHO sessions, a ‘hub and spoke’ model, where online wide, multi-disciplinary communities of practice take part in case-based learning and discussion^20^:

‘Our hospice has an education team and had the resources to run virtual learning and support sessions. [. . .] It would be great to see collaboration between the hospice and primary care in future education offers.’ (Respondent 162, General Practitioner in training, England)

In addition to formal training, participants described opportunity for informal training through closer working relationships with specialist palliative care colleagues. This was commonly described in areas where specialist palliative care teams had adopted more remote approaches during the pandemic.

‘Collaboration between the specialist palliative care teams and community teams. We worked closely together, teaching and discussing individual patients.’ (Respondent 524, Advanced Nurse Practitioner, England)

## Discussion

### Main findings

Individuals from primary healthcare teams reported a range of rapid service developments, such as changes in prescribing patterns and alterations to working hours, deemed necessary to meet increased need for community end-of-life care as a result of the COVID-19 pandemic. This was a challenging time in primary care, which led to community nurses taking greater responsibility in most areas of care including symptom control and the provision of support to family members. As modes of working moved to more virtual consultations, community nurses reported feelings of abandonment and general practitioners reported a sense of moral distress with reduced face-to-face contact with patients at the end-of-life. Working hours changed to meet rising demands for care at home through a mainly ‘ad hoc’ approach, and there was a significant emotional impact.^13^ Despite these challenges, primary care respondents to this survey could report positive changes. Greater flexibility in systems and processes and new opportunities for more responsive ways of working through the increased use of technology were described as beneficial. Participants reported a range of opportunities to strengthen interprofessional relationships across primary care and with specialist palliative care colleagues, through inter-disciplinary training as well as collaborative approaches to patient care.

### Strengths and limitations

This survey provides valuable insights into the role of primary healthcare, with a focus on the service changes and innovations that were considered to have worked well. The findings are relevant to the design of future service delivery models and policy during the next phases of the pandemic and beyond.

This is a survey of professional experiences and captures the views of professionals from across the UK, and more international primary care focussed research is necessary. The survey findings are limited by the response rate. The target number of responses was achieved, but the response rate was low amongst general practitioners. This may have been due to the timing of the survey or because there were a large number of other surveys seeking the views of general practitioners about other areas of practice during COVID-19. Furthermore, the findings are likely to reflect the views of primary care professionals with an active role or interest in palliative care and may not be representative of the wider population of primary care professionals.

### What this study adds?

Very little research has been conducted internationally into the response of primary healthcare services in the delivery of end-of-life care during previous pandemics,^11^ and it has received little focus during COVID-19. This study therefore addresses an important gap in the evidence by focussing on the experiences of primary healthcare professionals during the first phase of the COVID-19 pandemic, specifically the innovations and changes in service design that were perceived to be beneficial.

The number of deaths in the UK increased by 15% during 2020 compared to the previous 5-year average,^21–23^ and there was a marked shift in place of death to community settings.^9^ Recognition that pre-existing barriers to the provision of end-of-life care in the community needed to be addressed urgently at both an organisational level and amongst individual professionals led to rapid changes. This is described as an ‘unfreeze’ of the status quo and ‘change’ in Lewin’s behavioural change theory.^17,24^ Many of the system changes described align with longstanding policy objectives in the United Kingdom, including more collaborative, cross-boundary, end-of-life care and the increased use of technology such as virtual consultations and shared patient records.^25,26^ These changes were supported during the peaks of the COVID-19 pandemic by National Health Service incident response ‘Command and Control’ structures, designed to ensure that the rapid changes needed in healthcare organisations could be implemented at pace.^27^ Further work is urgently required to understand not only which changes led to improvements in community end-of-life care, and why, but how these positive changes can be sustained into the future (the ‘refreeze’^24^)

**Figure 1.**
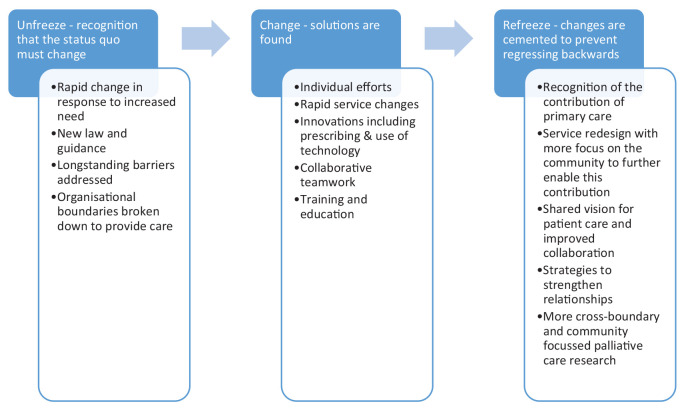
Summary of service changes and innovation in primary care end of life care during the COVID-19 pandemic, informed by Lewin’s (unfreeze-change-refreeze) model of behavioural change.

This analysis specifically focusses on innovations and service changes in primary care that were perceived to be beneficial. The positive descriptions of collaborative relationships across organisational boundaries, including virtual team meetings with colleagues from primary and specialist palliative care, contrast with the tensions described in the first analysis.^13^ Participants reported that team relationships were strengthened during COVID-19 with shared goals for the care of a patient. Collaborative relationships were enhanced through opportunities for joint training and education. More research is needed to understand how, when and why these relationships thrive and improve patient care. This must include research into how technology can most effectively improve collaborative teamwork and patient care. A summary of service changes informed by Lewin’s ‘unfreeze-change-refreeze’ model of behavioural change is provided in Figure 1.

The key role of primary care in the provision of end of life care during COVID-19 was highlighted in clinical prioritisation guidance at the start of the pandemic,^28,29^ but has been largely overlooked in palliative care research during the pandemic to date.^30,31^ Universal palliative and end-of-life care remains a pressing concern globally.^32^ As system leaders and policy makers work to embed learning from the reactive changes that occurred during COVID-19 (the ‘unfreeze’ and ‘change’) into future service design (the ‘refreeze’), strategies are required in order to enable both the efforts of motivated individuals and the contribution of primary care teams, alongside colleagues from specialist palliative care, to achieve the shared goal of universal palliative care for all.

## Conclusion

This study provides insights from primary healthcare teams into the individual efforts and service changes that were perceived to be beneficial through the first phase of the COVID-19 pandemic. The pivotal role of primary care in ensuring the global ambition of universal palliative care requires much more attention in future research, service design and policy. As international healthcare systems move to a period of restoration following the first phases of the COVID-19 pandemic, there is a need to ensure learning from rapidly implemented service changes. A once in a generation opportunity has arisen to incorporate cross-boundary service changes and innovations, implemented rapidly at the time of crisis into future service delivery. These include the use of technology, to facilitate more collaborative working, improved access to specialist palliative care and provision of palliative care in primary care settings. Future research should focus on which service changes and innovations provide the most benefits, who for, and how, within the context of increased patient need and complexity in the community.

## Supplemental Material

sj-pdf-1-pmj-10.1177_02692163211049311 – Supplemental material for Service change and innovation in community end-of-life care during the COVID-19 pandemic: Qualitative analysis of a nationwide primary care survey Click here for additional data file. Supplemental material, sj-pdf-1-pmj-10.1177_02692163211049311 for Service change and innovation in community end-of-life care during the COVID-19 pandemic: Qualitative analysis of a nationwide primary care survey by Sarah Mitchell, Madeleine Harrison, Phillip Oliver, Clare Gardiner, Helen Chapman, Dena Khan, Kirsty Boyd, Jeremy Dale, Stephen Barclay and Catriona R Mayland in Palliative Medicine
