# Supplementary material for: Service change and innovation in community end-of-life care during the COVID-19 pandemic: Qualitative analysis of a nationwide primary care survey
Source: Palliat Med. 2021 Dec 17;36(1):161–70. doi: 10.1177/02692163211049311 (PMC8796165; doi:10.1177/02692163211049311)
Supplement: sj-pdf-1-pmj-10.1177_02692163211049311 – Supplemental material for Service change and innovation in community end-of-life care during the COVID-19 pandemic: Qualitative analysis of a nationwide primary care survey [file sj-pdf-1-pmj-10.1177_02692163211049311.pdf]

Survey of the role and response of primary healthcare services in the delivery of palliative and end-of-life care during COVID-19

Thank you for agreeing to take part in this survey. The aim of this study is to capture and learn from the experiences of general practitioners (GPs) and community nurses in the delivery of end-of-life care in the community through the COVID-19 pandemic. There has been almost no research in this area during past pandemics.

Please complete the survey as soon as possible so that we can analyse and share the results. The survey will close on 30th September.

Throughout the survey, "palliative care" is defined as an approach to care focussed on improving quality of life for people affected by life-threatening conditions, by early identification and management of physical, psychological, social and spiritual concerns (World Health Organisation), rather than as a specialist service. Specialist services are specifically referred to as Specialist Palliative Care services and hospice services.

The questionnaire will take 15-20 minutes to complete

There are five short sections, followed by a section for longer free text answers. No questions are compulsory. Thank you very much for taking part.

*Required

1. Email *

____

2. I give consent for the use of anonymised quotes in reports of the survey results

Mark only one oval.

- ( ) Yes
- ( ) No

Skip to question 3

Section 1: About you and where you work

Please provide some details about your job role and where you work, and consent for the use of your responses

3. 1.1. Which country do you work in?

Mark only one oval.

- ( ) England
- ( ) Scotland
- ( ) Wales
- ( ) Northern Ireland

4. 1.2. In which Clinical Commissioning Group / Health Board area do you work?

____

5. 1.3. What type of area do you work in mainly?

Mark only one oval.

- ( ) Urban
- ( ) Rural
- ( ) Innercity
- ( ) Mixed urban and rural

6. 1.4. FOR DOCTORS: Your role (please tick all that apply)

Tick all that apply.

- [ ] GP partner
- [ ] Sessional GP
- [ ] GP in training
- [ ] Foundation year doctor in general practice
- [ ] GP with a Special Interest or Extended Role in Palliative Care
- [ ] Residential care home GP
- [ ] Nursing care home GP
- [ ] Out of Hours GP

7. 1.5. FOR NURSES AND COMMUNITY STAFF: Your role (please select)

Tick all that apply.

- [ ] Community healthcare assistant/Support Worker
- [ ] Community Staff Nurse
- [ ] Deputy District Nurse Team Leader
- [ ] District Nurse
- [ ] Advanced Nurse Practitioner
- [ ] Community Matron
- [ ] Nurse Consultant

8. 1.6. For ALL: Please indicate whether you have any extra training or experience in palliative care

Tick all that apply.

- [ ] Diploma in Palliative Care / Palliative Medicine
- [ ] MSc
- [ ] PhD / MD
- [ ] Experience of working in a hospice
- [ ] Experience of working in a specialist palliative care team (not hospice)

9. 1.7. FOR DOCTORS: What is your practice list size?

____

____

____

____

____

10. 1.8. FOR DOCTORS: Which of the following does your practice usually provide (please select all that apply)?

Tick all that apply.

- [ ] Practice has a palliative care register
- [ ] Practice has regular palliative care meetings involving the practice team
- [ ] Practice has multi-disciplinary palliative care meetings with other colleagues e.g. community nurses / specialist palliative care team
- [ ] None of the above

11. 1.9. FOR NURSES: Which of the following does your service usually provide? (there is a question about service developments through COVID-19 in a later section)

Tick all that apply.

- [ ] 24/7 visiting service
- [ ] Out of hours on call service
- [ ] 9am-5pm services only
- [ ] Subcutaneous medications (including specialist medications)
- [ ] Syringe drivers
- [ ] IV antibiotics in the community
- [ ] IV blood transfusions in the community
- [ ] Paracentesis
- [ ] Prescribing
- [ ] Transcribing of prescribed medications

12. 1.10. What local services do you usually work with to deliver palliative and end-of-life care?

Tick all that apply.

- [ ] Community support workers / social prescribing / voluntary sector
- [ ] Intensive home nursing service / Hospice at home
- [ ] 24/7 palliative care hub or other 24/7 helpline
- [ ] Community specialist palliative care team
- [ ] Local hospice specialist palliative care team
- [ ] Local hospital specialist palliative care team e.g. phone communication, handover, information sharing

Section 2: Caring for dying patients during the COVID-19 pandemic

This section is about the types of patients that you have provided end-of-life care to in the community during the COVID-19 pandemic.

13. 2.1. Have you cared for any patients in the community who have died with confirmed (by test) COVID-19?

Mark only one oval.

- Yes
- No

14. 2.2. Have you cared for any patients who have died with suspected COVID-19 (untested but with clinical diagnosis / symptoms)?

Mark only one oval.

- Yes
- No

15. 2.3. Have you been involved in providing end-of-life care at home for patients who do not have COVID-19 or suspected COVID-19 through the pandemic?

Mark only one oval.

- A lot more than usual
- A bit more than usual
- About the same as usual
- A bit less than usual
- A lot less than usual

Section 3: Changes in general practice / community nursing palliative care in response to COVID-19

This section starts with questions about changes in your personal practice, and goes on to ask more about how your services have changed to deliver palliative and end-of-life care in response to COVID-19.

16. 3.1. Has your personal involvement in palliative care changed?

Mark only one oval.

- ( ) Yes
- ( ) No
- ( ) Stayed the same

17. 3.2. Please state whether your role in the following areas of palliative care has changed during the COVID-19 pandemic

Mark only one oval per row.

	doing a lot less	doing less	stayed the same	doing more	doing a lot more	not my role
• Advance care planning	( )	( )	( )	( )	( )	( )
• Anticipatory prescribing	( )	( )	( )	( )	( )	( )
• Symptom management	( )	( )	( )	( )	( )	( )
• Support for family members and carers	( )	( )	( )	( )	( )	( )
• Bereavement support	( )	( )	( )	( )	( )	( )
• Death verification	( )	( )	( )	( )	( )	( )
• Death certification	( )	( )	( )	( )	( )	( )
• Palliative care for patients who do not have COVID-19	( )	( )	( )	( )	( )	( )
• Collaborative working with specialist palliative care teams	( )	( )	( )	( )	( )	( )

18. 3.3. Have you had particular challenges or issues in any of the following aspects of palliative care provision? (please select all that apply)

Tick all that apply.

- [ ] Advance care planning
- [ ] Symptom management including COVID-19 specific symptoms
- [ ] Medicines supplies
- [ ] Bereavement support
- [ ] Death verification
- [ ] Death certification
- [ ] Palliative care for patients who do not have COVID-19
- [ ] Collaborative working with specialist palliative care teams
- [ ] Care and support for caregivers

19. 3.4. Have there been any guidelines (local or national) that you have found particularly helpful?

____
____
____
____
____

20. 3.5. Have your working hours changed in order to deliver end of life care during COVID-19?

Mark only one oval.

- ( ) Yes
- ( ) No

21. 3.6. If yes, please provide details of the impact of providing end of life care during the COVID-19 pandemic on your working hours and whether this has been a formal or informal change

22. 3.7. Have there been any changes in the organisation of your team in order to provide end of life care during the COVID-19 pandemic?

Mark only one oval.

- Yes
- No

23. 3.8. Please provide details about how the organisation of your team has changed.

24. 3.9. Have any of the following services been developed in your area in response to the COVID-19 pandemic?

Tick all that apply.

- [ ] 24/7 community nursing visiting service
- [ ] Out of hours community nursing on call service
- [ ] Extended hours community nursing beyond a 9am-5pm service
- [ ] Subcutaneous medications (including specialist medications)
- [ ] Syringe drivers
- [ ] IV antibiotics in the community
- [ ] IV blood transfusions in the community
- [ ] Paracentesis in the community
- [ ] Prescribing
- [ ] Transcribing of prescribed medications
- [ ] Carer administration of end of life care drugs
- [ ] 24/7 specialist palliative care team visiting service
- [ ] Out of hours specialist palliative care team on call service

Other: [ ] ______________________

Section 4: Changes in how you support patients at the end of life and their families

This section is about how you have provided care for dying patients at home, including the use of technology

25. 4.1. How have your consultations with patients at the end-of-life and their carers changed? (please select)

Mark only one oval per row.

	Doing a lot less	Doing less	Stayed the same	Doing more	Doing a lot more	Not my role
Home visits	◯	◯	◯	◯	◯	◯
Telephone consultations	◯	◯	◯	◯	◯	◯
Virtual consultations	◯	◯	◯	◯	◯	◯
Face-to-face consultations in surgery	◯	◯	◯	◯	◯	◯

26. 4.2. Are you using virtual technologies with colleagues to provide end-of-life care (please select)

Mark only one oval.

- A lot more
- Slightly more
- About the same
- Slightly less
- Much less

27. 4.3. Please add any comments about changes in consultations or the use of technology

Section 5: Patients in care homes (including nursing homes)

This section considers care for patients in care homes. Please complete if you are involved in providing care in these settings

28. 5.1. Do you provider general practice / community nursing services in care homes?

Mark only one oval.

- Yes (please complete the rest of this section)
- No (please move on to section 6)

29. 5.2. How many care homes do you / your practice / your community nursing team provide care for?

30. 5.3. What types of care home do you provide care for?

Mark only one oval.

- Nursing homes
- Specialist nursing homes
- Residential care homes
- Community hospital
- Assisted living facilities
- Mental health care (EMI) homes
- Other: ____________

31. 5.4. Has the number of patients in care homes needing end-of-life care changed during the COVID-19 pandemic?

Mark only one oval.

- Increased
- Stayed the same
- Decreased

32. 5.5. Has your face-to-face contact with patients in care homes changed?

Mark only one oval.

- Increased
- Stayed the same
- Decreased

33. 5.6. Has your use of telephone and video consultations with patients in care homes changed?

Mark only one oval.

- Increased
- Stayed the same
- Decreased

Section 6: Comments, challenges and opportunities

This section is for you to provide any details about your experiences of providing end-of-life care in the community through the COVID-19 pandemic. Please include anything that you would like to or that you think are important experiences to learn from. This might include any reflections on providing care to patients at home, working in care homes, the impact on you, and care of families and carers including bereavement.

34. 6.1. Please share any personal reflections on your experiences of the provision of end-of-life care in the community and in care homes during the COVID-19 pandemic

35. 6.2. Is there anything that you think has worked particularly well? Please include any changes or innovations that you would like to see continue beyond COVID-19

36. 6.3. Is there anything that you feel has not worked very well?

37. 6.4. What do you think will be the biggest challenges in the future delivery of palliative and end-of-life care in the community over the next 12 months? Please detail

38. 6.5. What do you think are the opportunities?

39. 6.6. Have there been any other learning points or particularly memorable experiences of providing end-of-life care during COVID-19 that you are willing to share? Please provide details

Next steps

Please let us know whether you would be willing to take part in a follow-up telephone or online interview about your experiences of end-of-life care during COVID-19

40. 7.1. Would you be interested in taking part in a follow-up interview about your experiences of end-of-life care provision during COVID-19?

Mark only one oval.

- Yes I would be interested in taking part in an interview, please contact me by email
- No I would not like to take part in an interview

41. 7.2. I would like to receive a copy of the survey results via email

Mark only one oval.

- Yes please
- No thank you

This content is neither created nor endorsed by Google.

Google Forms
